# Comparison of Opioid Prescribing by Dentists in the United States and England

**DOI:** 10.1001/jamanetworkopen.2019.4303

**Published:** 2019-05-24

**Authors:** Katie J. Suda, Michael J. Durkin, Gregory S. Calip, Walid F. Gellad, Hajwa Kim, Peter B. Lockhart, Susan A. Rowan, Martin H. Thornhill

**Affiliations:** 1 Center of Innovation for Complex Chronic Healthcare, Edward Hines Jr Veterans Administration Hospital, Chicago, Illinois; 2 College of Pharmacy, University of Illinois at Chicago; 3 School of Medicine, Washington University in St Louis, St Louis, Missouri; 4 Center for Health Equity Research and Promotion, Pittsburgh Veterans Administration Healthcare System, Pittsburgh, Pennsylvania; 5 School of Medicine, University of Pittsburgh, Pittsburgh, Pennsylvania; 6 Center for Clinical and Translational Science, University of Illinois at Chicago; 7 Department of Oral Medicine, Carolinas Medical Center, Charlotte, North Carolina; 8 College of Dentistry, University of Illinois at Chicago; 9 School of Clinical Dentistry, University of Sheffield, Sheffield, United Kingdom

## Abstract

**Importance:**

The United States consumes most of the opioids worldwide despite representing a small portion of the world’s population. Dentists are one of the most frequent US prescribers of opioids despite data suggesting that nonopioid analgesics are similarly effective for oral pain. While oral health and dentist use are generally similar between the United States and England, it is unclear how opioid prescribing by dentists varies between the 2 countries.

**Objective:**

To compare opioid prescribing by dentists in the United States and England.

**Design, Setting, and Participants:**

Cross-sectional study of prescriptions for opioids dispensed from outpatient pharmacies and health care settings between January 1 and December 31, 2016, by dentists in the United States and England. Data were analyzed from October 2018 to January 2019.

**Exposures:**

Opioids prescribed by dentists.

**Main Outcomes and Measures:**

Proportion and prescribing rates of opioid prescriptions.

**Results:**

In 2016, the proportion of prescriptions written by US dentists that were for opioids was 37 times greater than the proportion written by English dentists. In all, 22.3% of US dental prescriptions were opioids (11.4 million prescriptions) compared with 0.6% of English dental prescriptions (28 082 prescriptions) (difference, 21.7%; 95% CI, 13.8%-32.1%; *P* < .001). Dentists in the United States also had a higher number of opioid prescriptions per 1000 population (35.4 per 1000 US population [95% CI, 25.2-48.7 per 1000 population] vs 0.5 per 1000 England population [95% CI, 0.03-3.7 per 1000 population]) and number of opioid prescriptions per dentist (58.2 prescriptions per dentist [95% CI, 44.9-75.0 prescriptions per dentist] vs 1.2 prescriptions per dentist [95% CI, 0.2-5.6 prescriptions per dentist]). While the codeine derivative dihydrocodeine was the sole opioid prescribed by English dentists, US dentists prescribed a range of opioids containing hydrocodone (62.3%), codeine (23.2%), oxycodone (9.1%), and tramadol (4.8%). Dentists in the United States also prescribed long-acting opioids (0.06% of opioids prescribed by US dentists [6425 prescriptions]). Long-acting opioids were not prescribed by English dentists.

**Conclusions and Relevance:**

This study found that in 2016, dentists in the United States prescribed opioids with significantly greater frequency than their English counterparts. Opioids with a high potential for abuse, such as oxycodone, were frequently prescribed by US dentists but not prescribed in England. These results illustrate how 1 source of opioids differs substantially in the United States vs England. To reduce dental opioid prescribing in the United States, dentists could adopt measures similar to those used in England, including national guidelines for treating dental pain that emphasize prescribing opioids conservatively.

## Key Points

**Question:**

How do opioid prescribing patterns differ between dentists in the United States and dentists in England?

**Findings:**

In this cross-sectional study of opioid prescribing by dentists in 2016, the proportion of dental prescriptions that were opioids was 37 times greater in the United States than in England.

**Meaning:**

In light of similar oral health and dentist use between the 2 countries, it is likely that opioid prescribing by US dentists is excessive and could be reduced.

## Teaser

This cross-sectional study compares the rate of opioid prescriptions by dentists dispensed from outpatient pharmacies and health care settings in the United States and England in 2016.

## Introduction

Dentists are among the most frequent prescribers of opioids in the United States, second after family physicians.^1,2^ While per capita prescribing of opioids is decreasing nationally,^3^ dental prescribing rates are increasing.^4^ Studies in the United States have shown that dentists recommend and prescribe opioids over nonsteroidal anti-inflammatory drugs, in greater quantities, and for longer than necessary to control dental pain.^5,6^ An estimated 1 million opioid pills prescribed following tooth extractions remain unused in the United States.^7^ Furthermore, dentists are responsible for one-third of opioid prescriptions to adolescents, a vulnerable population for opioid misuse.^1,8^

Worldwide, opioid use varies significantly by country.^9^ The United States consumes most of the global opioid supply despite representing only 4% of the world’s population.^9^ Compared with the United States, England has lower overall opioid prescribing rates.^9,10^ One of the reasons opioid prescribing may be lower is because of differing prescribing patterns among dentists. Dental care is subsidized as part of the public benefit in the United Kingdom’s (UK) National Health Service (NHS), while 23% of the US population does not have dental insurance.^11^ To inform the debate about dental opioid prescribing, we used nationally representative data to compare opioid prescribing between dentists in the United States and England.

## Methods

This study was a population-level analysis of nationally representative databases of prescriptions dispensed from outpatient pharmacies in the United States and England from January 1 to December 31, 2016. This study followed the Strengthening the Reporting of Observational Studies in Epidemiology (STROBE) reporting guideline. The University of Illinois at Chicago investigational review board deemed that this study was exempt from review and informed consent. Systemic opioids dispensed from community and mail service pharmacies and outpatient clinics were included. The opioid class was defined as products containing codeine, fentanyl, hydrocodone, hydromorphone, levorphanol, meperidine, methadone, morphine, oxycodone, oxymorphone, tapentadol, tramadol, pentazocine-naloxone, and butorphanol single-entity formulations (not combined with naloxone). Long-acting opioids included buprenorphine, levorphanol, methadone, fentanyl transdermal patches, and controlled- or extended-release morphine, oxycodone, oxymorphone, and hydromorphone.

### Data Sources

We obtained US data from IQVIA LRx, which captures 85% of all outpatient prescriptions. With the exception of the Veterans Health Administration, data in LRx contain all patients regardless of payer, including commercially insured, Medicare, Medicaid, and cash pay. We obtained prescribing data for England from the NHS Digital Prescription Cost Analysis. Data from NHS were only available for England (84.2% of the UK population)^12^ and did not include Scotland, Wales, or Northern Ireland. Prescribing by dentists in England is restricted to a national formulary as listed in the Dental Practitioners Formulary, part of the British National Formulary.^13^ All outpatient prescriptions prescribed by dentists in the United States and England were included; any prescriptions with missing values (eg, missing number of days supplied) were not included in our analysis.

### Outcomes

We measured 3 outcomes on opioid prescribing by dentists: (1) an overall number of opioid prescriptions, (2) the proportion of all prescriptions that included opioids, and (3) prescribing rates. Rates were adjusted for population size as reported by the US Census Bureau and UK Office for National Statistics. Rates were similarly adjusted for annual numbers of licensed dentists as defined by the American Dental Association (n = 196 441) and the UK General Dental Council (n = 24 007).

### Statistical Analysis

To assess differences in proportions of opioid prescriptions and specific drugs by country, χ^2^ and Fisher exact tests were applied as appropriate. A 2-sided *P *value less than or equal to .05 was considered significant. We used SAS statistical software version 9.4 (SAS Institute Inc) for statistical analyses. Results are reported with Poisson exact confidence intervals.

## Results

In 2016, dentists prescribed more than 11.4 million opioid prescriptions in the United States and 28 082 opioid prescriptions in England. The proportion of all dental prescriptions written for opioids was 37 times greater in the United States than in England (22.3% of US dental prescriptions were for opioids vs 0.6% of English dental prescriptions; difference, 21.7%; 95% CI, 13.8%-32.1%; *P* < .001). Dentists in the United States also had higher prescribing rates when values were adjusted for population (35.4 per 1000 US population [95% CI, 25.2-48.7] vs 0.5 per 1000 England population [95% CI, 0.03-3.7]) and number of dentists (58.2 per clinician [95% CI, 44.9-75.0] vs 1.2 per clinician [95% CI, 0.2-5.6]) (Table 1).

**Table 1.** Dental Prescribing Rates and Frequencies in the United States and England, 2016

Prescribing Outcomes	United States	England
Dental opioid prescriptions, No.	11 440 198	28 082
Population-based opioid prescribing rate, No. of prescriptions per 1000 population (95% CI)	35.4 (25.2-48.7)	0.5 (0.03-3.7)
Clinician-based opioid prescribing rate, No. of prescriptions per dentist (95% CI)	58.2 (44.9-75.0)	1.2 (0.2-5.6)

There were also differences in the drugs prescribed by country. In England, the only opioid analgesic prescribed by dentists was the codeine derivative dihydrocodeine. A much wider range of opioids were prescribed by US dentists (Figure). Hydrocodone-based opioids accounted for most (62.3%) of US dental opioid prescribing, followed by codeine (23.2%), oxycodone (9.1%), and tramadol (4.8%) (Table 2). While infrequent, prescribing of long-acting opioids by US dentists did occur (0.06% of opioids prescribed [6425 prescriptions]). Long-acting and high-potency opioids (eg, oxycodone, meperidine) were not prescribed by dentists in England.

**Figure.**
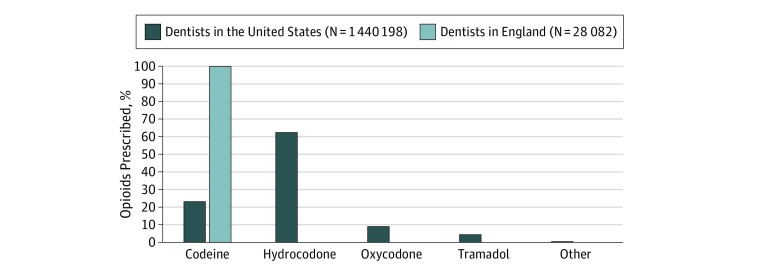
Opioids Prescribed by Dentists in the United States and England, 2016

**Table 2.** Dental Prescribing Rates and Frequencies in the United States and England by Drug, 2016

Prescription	United States	England
Codeine		
Dental codeine prescriptions, No.	2 657 486	28 082
Population-based codeine prescribing rate, No. of prescriptions per 1000 population (95% CI)	8.2 (4.1-15.8)	0.5 (0.03-3.7)
Clinician-based codeine prescribing rate, No. of prescriptions per dentist (95% CI)	13.5 (7.7-22.2)	1.2 (0.2-5.6)
Hydrocodone		
Dental hydrocodone prescriptions, No.	7 126 867	0
Population-based hydrocodone prescribing rate, No. of prescriptions per 1000 population (95% CI)	22.0 (13.8-33.3)	0 (0-3.7)
Clinician-based hydrocodone prescribing rate, No. of prescriptions per dentist (95% CI)	36.3 (26.1-49.8)	0 (0-3.7)
Oxycodone		
Dental oxycodone prescriptions, No.	1 044 611	0
Population-based oxycodone prescribing rate, No. of prescriptions per 1000 population (95% CI)	3.2 (1.1-8.8)	0 (0-3.7)
Clinician-based oxycodone prescribing rate, No. of prescriptions per dentist (95% CI)	5.3 (2.2-11.7)	0 (0-3.7)
Tramadol		
Dental tramadol prescriptions, No.	543 630	0
Population-based tramadol prescribing rate, No. of prescriptions per 1000 population (95% CI)	1.7 (0.2-5.6)	0 (0-3.7)
Clinician-based tramadol prescribing rate, No. of prescriptions per dentist (95% CI)	2.8 (0.6-7.2)	0 (0-3.7)
Other opioids^a^		
Other opioid prescriptions by dentists, No.	67 604	0
Population-based prescribing rate of other opioids, No. of prescriptions per 1000 population (95% CI)	0.2 (0.03-3.7)	0 (0-3.7)
Clinician-based prescribing rates of other opioids, No. of prescriptions per dentist (95% CI)	0.3 (0.03-3.7)	0 (0-3.7)

^a^ The category of other opioids includes levorphanol (n = 2), opium (n = 8), oxymorphone (n = 454), butorphanol (n = 176), buprenorphine (n = 338), methadone (n = 579), tapentadol (n = 695), pentazocine (n = 1463), fentanyl (n = 1466), morphine (n = 4430), hydromorphone (n = 9774), and meperidine (n = 48 219).

## Discussion

Compared with English dentists, US dentists’ prescribing of opioids is substantial. This includes opioids with a higher potential for diversion or abuse (eg, oxycodone, long-acting opioids). The significantly higher opioid prescribing occurs despite similar patterns of receiving dental care by children and adults, no difference in oral health quality indicators, including untreated dental caries and edentulousness, and no evidence of significant differences in patterns of dental disease or treatment between the 2 countries.^14,15,16,17^ Although there are greater oral health inequalities associated with education level and income in the United States, the overall oral health of US and UK residents is very similar.^14,15,16,17^ To our knowledge, this is the first study comparing dental opioid prescribing practices between countries.

Several studies have demonstrated that oral opioids do not provide superior pain control compared with nonopioid analgesics for acute and chronic pain.^18^ Systematic reviews and randomized clinical trials of acute oral pain found that patients who received acetaminophen combined with ibuprofen reported pain relief that was noninferior or superior to regimens with an opioid and nonopioid combination analgesic.^19,20^ Opioid-containing regimens were also associated with the highest risk of adverse events.^20^ It has been estimated that more than half of opioid pills prescribed for oral pain remain unused, and unused opioids have been shown to be a source of nonmedical opioid use.^7,21^ Additionally, access to dental care in the United States has been associated with higher rates of opioid abuse via increased opioid availability, leading to patients with substance use disorder targeting dentists.^3,21^

Several efforts are underway to improve opioid prescribing in the United States. The White House has created a commission and released guidance on combating the opioid crisis. The Centers for Disease Control and Prevention have created guidelines and other resources to assist health care professionals with opioid management.^18^ State-level policies, like prescription drug monitoring programs, now exist in every US state except Missouri. Despite national and state guidelines on opioid management and online material from the American Dental Association to facilitate safe opioid prescribing, there are no guidelines for oral pain available in the United States. In England, however, national guidelines are available for oral pain and recommend early definitive dental treatment (eg, surgical drainage) as the best treatment for most dental pain.^22^ When analgesics are required, these guidelines recommend nonsteroidal anti-inflammatory drugs as preferable to opioids. However, the major factor likely driving the difference in dentist opioid prescribing practices between the countries is that English dentists prescribe according to a medication formulary; dihydrocodeine is the only opioid included.^13,22^ In the United States, dentists are not restricted to certain opioids (any prescription opioid can be prescribed by a dentist). Thus, US dentists and medical professionals are able to prescribe the same medications, and there are no restrictions for dentist prescribing of a specific medication. It has also been reported that US dentists have an overperception of the level of pain associated with dental procedures compared with what is actually experienced by their patients.^23^ Other factors associated with high prescribing of opioids in the United States include pharmaceutical marketing, regulatory initiatives to treat pain, abundant supply of opioids, and patient perception and satisfaction.^18,24,25,26^

### Limitations

Our analysis is not without limitations. The English data are limited to patients receiving medications through the NHS. However, dentists are required to prescribe medications consistent with the dental formulary regardless of payer, and opioid prescribing outside the NHS formulary in the United Kingdom is infrequent (0.1%).^10^ Clinician-based rates were calculated with the number of licensed dentists, including dentists not actively practicing. Thus, the clinician-based rates are a conservative estimate. Patient- and visit-level data were not available. Therefore, we are unable to determine the appropriateness of prescribing. However, the substantial differences observed strongly suggest that opioid prescribing by US dentists is excessive and could be contributing to the opioid epidemic. Therefore, strategies to aid dentists in the judicious prescribing of opioids should be implemented. Individual dental practices should implement local pain management guidelines that recommend nonopioid analgesics (unless contraindications are present) and query their local prescription drug monitoring program (PDMP) before prescribing controlled substances. When opioids are indicated, dentists should prescribe only short-acting, low-potency opioids for the shortest duration consistent with anticipated postprocedural pain. Similar to England, public health and professional organizations should provide oral pain guidelines, provide educational programming focused on the treatment of oral pain, and restrict the scope, strength, and duration of opioids that can be prescribed by dentists.

## Conclusions

While the opioid epidemic has not been isolated to the United States, opioid prescriptions and opioid-related deaths in the United States far exceed reports of other countries, including the United Kingdom.^27^ These results illustrate how one potential source of opioids differs substantially in the United States vs England and highlights the need for efforts to reduce US dental opioid prescribing. Future research should determine factors associated with high opioid prescribing by dentists and determine effective strategies to improve opioid prescribing for oral health conditions. Appropriate opioid prescribing in persons without dental insurance and of lower socioeconomic status should also be assessed to determine whether accessibility to oral health care is associated with overprescribing of opioids. Because definitive dental treatment is not readily available in most urgent care centers and emergency departments, differences in opioid prescribing for oral pain outside of dentistry should also be assessed. Curtailing opioid prescribing will require a multifaceted approach by agencies and educational programs directed at dentists and their patients. This may also involve the introduction of national or specialty-specific guidelines and consideration of formularies that limit the scope of opioid prescribing by dentists.
